# Next generation sequencing yields the complete mitogenome of captive forest musk deer, *Moschus berezovskii* (Ruminantia: Moschidae)

**DOI:** 10.1080/23802359.2018.1462670

**Published:** 2018-04-23

**Authors:** Chao Yang, Jie Tang, Kun Bian, Li-Juan Suo, Hao Yuan, Yan Wang, Yuan Huang

**Affiliations:** a Shaanxi Institute of Zoology, Xi’an, China;; b School of Life Sciences, Shaanxi Normal University, Xi’an, China

**Keywords:** *Moschus berezovskii*, mitogenome, phylogeny, subspecies

## Abstract

*Moschus berezovskii* is an endangered species, but its captive populations are valuable on musk secretions in traditional Chinese medicine and perfume manufacture. The mitogenome of *M. berezovskii* was 16,353 bp in size. Stop codons in 13 PCGs were all typical types except incomplete stop codon T for *COX3*, *ND2* and *ND4*, and TA for *ND3.* No tandem repeat was found in control region. Phylogenetic analysis indicated that Moschidae has the closest relationship with Bovidae. We supported that *M. berezovskii* should be categorized into two subspecies, and suggested that the status of *M. chrysogaster* JQ608470 should be further investigated.

Forest Musk Deer, *Moschus berezovskii* was listed as a threatened species in the Red List (IUCN 2017) and categorized as a first-degree national protected species in China. Once abundant, poaching and habitat loss had led to a dramatic decrease in wild population numbers (Gang et al. 2015) prompting to establish captive breeding populations for musk secretions. Nevertheless, during the long process of captive breeding, historical records about the genetic backgrounds of many captive populations were either lost or incomplete. Therefore, understanding the genetic diversity and phyletic evolution of the captive populations are the two most important tasks for excellent provenance selection (Peng et al. 2008).

Sample (voucher no. LS018) of captive *M. berezovskii* was deposited in the animal specimens museum of Shaanxi Institute of Zoology, Xi’an, China. Genomic DNA was prepared in 150 bp paired-end libraries, tagged and subjected to the high-throughput Illumina Xten platform and yielded 19,970,960 Paired-End Raw Reads. Mapping against the complete mitogenome of *M. moschiferus* (GenBank: KT337321), high-quality reads were assembled using MITObim version 1.9 (Hahn et al. 2013). A total of 16,923 individual mitochondrial reads gave an average coverage of 154.7X. Comparing with the *M. moschiferus*, annotations were generated in MITOchondrial genome annotation Server (MITOS) (Bernt et al. 2013) and Geneious version 10.1.2.

The complete mitogenome sequence consists of 16,353 bp for *M. berezovskii* (GenBank: MH047347). The typical ATN (ATG or ATT or ATA) start codons are present in PCGs. TAA and AGA stop codons are used for most genes, with the exception of incomplete stop codon T for *COX3*, *ND2* and *ND4*, and TA for *ND3.* The two rRNA genes are 955 bp in *srRNA* and 1571 bp in *lrRNA*. The tRNA genes have the typical cloverleaf secondary structures except for the shortest *tRNA^Ser^(AGN)* which lacks the DHU arm. A tandem repeat was not found in 924-bp-long non-coding region (Kim et al. 2017).

For phylogenetic analyses of Moschidae, MrBayes ver. 3.2.2 (Ronquist et al. 2012) and RAxML (Stamatakis 2006) were used to reconstruct BI and ML tree, understanding the best partitioned scheme and optimal model analysed in Partitionfinder v1.1.1 (Lanfear et al. 2012) (models GTR + I+G and GTR + G). *Hippopotamus amphibius* (GenBank: AP003425) and *Camelus bactrianus* (GenBank: EF507798) were selected as outgroups. The phylograms obtained from BI and ML (data not shown) all strongly indicated that Moschidae was a sister group to Bovidae (Hassanin and Douzery 2003; Yang et al. 2013; Pan et al. 2015). In Moschidae, our analysis supported that *M. berezovskii* should be divided into two subspecies, and the captive *species* was most closely related to the *M. anhuiensis* and the wild species was most closely related to *M. chrysogaster* (Su et al. 1999; ). Extraordinarily, the *M. chrysogaster* JQ608470 was located in the top of the tree, but not belonged to the branch of (*M. chrysogaster* KP684123*, M. berezovskii* wild) (Figure 1).

**Figure 1.**
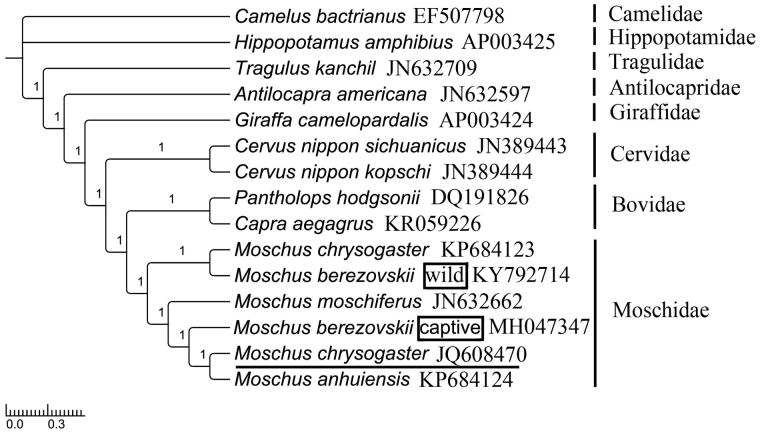
Topology of Bayesian tree for 15 species based on mitogenome PCGs sequences. GenBank accession numbers are indicated following species name (numbers on nodes are bootstrap values).
